# Bidirectional relationships between retention and health-related quality of life in Chinese mainland patients receiving methadone maintenance treatment

**DOI:** 10.1371/journal.pone.0179009

**Published:** 2017-06-06

**Authors:** Kaina Zhou, Duolao Wang, Hengxin Li, Xiaoli Wei, Juan Yin, Peifeng Liang, Lingling Kou, Mengmeng Hao, Lijuan You, Xiaomei Li, Guihua Zhuang

**Affiliations:** 1 Xi’an Jiaotong University Health Science Center, Xi’an, Shaanxi, China; 2 Liverpool School of Tropical Medicine, Pembroke Place, Liverpool, United Kingdom; 3 Xi’an Center for Disease Control and Prevention, Xi’an, Shaanxi, China; Centre for Addiction and Mental Health, CANADA

## Abstract

This study aimed to explore the bidirectional relationships between retention and health-related quality of life (HRQoL) in patients from mainland China receiving methadone maintenance treatment (MMT). This prospective cohort study recruited 1,212 eligible MMT patients from the two largest MMT clinics (one privately and another publicly funded) in Xi’an. This study started in March 2012 with a 2-year follow-up until March 2014. Retention was assessed by repeated terminations, past treatment duration, premature terminations, and follow-up treatment duration. HRQoL was evaluated using the Chinese (simple) short-form 36 health survey version 2 (SF-36v2) and the quality of life scale for drug addicts (QOL-DAv2.0). Linear and Cox regression analyses were used to explore relationships between retention and HRQoL. A general linear model was used to further examine the global effect of past treatment duration on HRQoL. Multivariate analyses showed that repeated terminations had no significant impact on HRQoL scores in MMT patients; however, past treatment time (year) influenced the SF-36v2PCS (*P* = 0.004): treatment for ≥4 years showed a lower SF-36v2PCS score (regression coefficient: -2.39; 95% confidence interval [CI]: -3.80, -0.97; *P* = 0.001) than treatment for <1 year. In addition, patients with an SF-36v2PCS score > 49 (hazard ratio: 0.83; 95% CI: 0.69, 0.98; *P* = 0.03) were 17% less likely to terminate MMT than those with scores of ≤49. In conclusion, retention and HRQoL tended to have a bidirectional relationship, which should be considered in the development of retention and health-management programs for patients with MMT.

## Introduction

Methadone maintenance treatment (MMT) is a harm-reduction program for people with opioid dependence; it involves the use of methadone as a synthetic agent to block the brain receptors affected by heroin and other opiates [1]. In China, MMT was initiated as a pilot program in 8 clinics assisting 1,029 drug users in 2004 and subsequently expanded to 761 clinics assisting 407,000 drug users by the end of 2013, aiding approximately 16% of domestic registered drug users [2,3]. A large body of evidence indicates that MMT can reduce drug addiction, drug-related harms, risk behaviors, crimes, and transmission of human immunodeficiency virus (HIV) and hepatitis C virus (HCV) [4,5]. Drug users receiving MMT show markedly improved personal relationships, enhanced social productivities, and better health-related quality of life (HRQoL) [6–9]. Given that MMT involves drug substitution and long-term administration, retention is the main factor that directly influences the treatment outcomes and a major indicator for the therapeutic effects of the program [10,11].

A long retention period is related to improved treatment outcomes, and the retention duration is considered the best predictor for the effects of MMT [11–13]. However, retention is suboptimal in most MMT programs, with retention rates ranging from 58% to 78% in China [14] and 3% to 94% worldwide [11]. Additionally, repeat dropout is common during the MMT process, which probably has adverse effects on treatment outcomes, especially patient-reported outcomes such as HRQoL. Many previous studies have reported that MMT positively affects HRQoL [9,15]; however, few studies have explored the influence of retention on HRQoL, particularly in patients with repeat terminations [16].

HRQoL is broadly conceptualized as individuals’ perception of their position in life in terms of their physical health, psychological health, social relationships, relationship with their environment, independence level, and personal beliefs [17]. It reflects many aspects of individuals’ daily lives and has been considered an important outcome measure in patients receiving MMT. Many published studies have demonstrated that MMT patients with good HRQoL have characteristics of strong social support, relatively independent income, few incidents of drug overdose and related complications (e.g., HIV or HCV infection, psychiatric comorbidity, and cutaneous abscess), and few incarcerations [18–20]. Regarding social support, we have previously explored the relationship between perceived social support and retention with the same patient population used in this study and found that good perceived social support is a strong predictor of retention [21]. However, this finding is insufficient to confirm a positive correlation between retention and HRQoL. From the viewpoint of MMT-participation behaviors, it is unclear whether patients with better HRQoL have longer retention periods [22].

To further our previous study [21], the present study aimed to identify bidirectional relationships between retention and HRQoL in patients from mainland China receiving MMT. We proposed 3 hypotheses: (a) patients with repeat terminations have poorer HRQoL than those with no terminations, (b) longer past treatment duration predicts better HRQoL, and (c) better HRQoL predicts less premature terminations.

## Methods

### Study design

This is a prospective cohort study. A 2-year follow-up was conducted on the basis of a cross-sectional survey.

### Participants and data collection

Patients admitted to the two largest MMT clinics (one funded privately and another funded publicly) in Xi’an, China, were recruited in the study. Inclusion criteria were age ≥ 18 years and fluency in Chinese. Patients with cognitive disorders or those who refused to provide written informed consent were excluded.

The study was started in March 2012, with a 2-year follow-up until March 2014. Data were collected in March 2012, including baseline information (i.e., pretreatment socio-demographics and drug use characteristics), retention, and HRQoL. The recruited MMT patients were subjected to individual face-to-face interviews by trained interviewers in a quiet and well-lit room.

### Instruments and study variables

#### Retention

Retention was the primary outcome of this study and was measured as the following 4 outcomes: (i) Repeated terminations, referring to the dropout history since the first MMT admission. (ii) Past treatment duration, representing the number of MMT participation days between the first MMT admission date and March 31, 2012. Data on both repeated terminations and past treatment duration were collected in the cross-sectional survey. (iii) Premature terminations, reflecting whether patients continued MMT after the 2-year follow-up; patients who were off methadone for 7 consecutive days were considered to have terminated treatment [23]. (iv) Follow-up treatment duration, indicating the time (days) from the start of MMT participation at baseline (April 1, 2012) to the occurrence of premature termination of MMT during the follow-up period, was calculated as time-to-event data.

#### HRQoL

HRQoL was the secondary outcome of this study and was measured using the short-form 36 health survey version 2 (SF-36v2) and the Quality of Life Scale for Drug Addicts (QOL-DAv2.0) in a cross-sectional survey.

*SF-36v2*. The Chinese (simple) SF-36v2 consists of 36 items and 8 multi-item scales [24]. The scales include physical functioning, limitations due to physical health problems (role physical), bodily pain, general health, vitality, social functioning, limitations due to emotional health problems (role emotional), and mental health. The 8 multi-item scales were aggregated into a physical component summary (PCS) and mental component summary (MCS), indicating physical and psychological HRQoL, respectively. The two summary components and 8 scales were scored using QualityMetric Health Outcomes^TM^ Scoring Software 4.0 [25], according to which a normal score was considered to be 50 ± 10 points (mean ± standard deviation). The summary components and scale scores ranged from 0 to 100, with higher scores representing better HRQoL. The original U.S. version of the SF-36v2 has good reliability and validity [24]. Cronbach’s α was 0.80 for PCS and 0.86 for MCS, both of which were calculated in our previous related study [26].

*QOL-DAv2*.*0*. The QOL-DAv2.0 consists of 40 items comprising 4 scales of physiology, psychology, society, and symptoms [27]. The 4 scales are scored according to the corresponding item scores, and the total score is calculated as a sum of the 4 scale scores, representing the HRQoL in drug addicts. All scores were standardized using the following formula: ([actual score—the lowest possible score]/[the highest possible score—the lowest possible score]) * 100 [28]. After standardization, each of the 4 scale scores and the total scores ranged from 0 to 100, with higher scores reflecting better HRQoL. Standardization makes it easier to compare the scores between different scales in order to understand the level of the total score. According to Wan et al. [27], the QOL-DAv2.0 has acceptable reliability and validity. Cronbach’s α was 0.88 for physiology, 0.93 for psychology, 0.80 for society, and 0.92 for symptoms, all of which were calculated in our previous related study [26].

### Data analyses

A database was built using Epidata 3.1. Two data managers double-entered the data to capture data-entry errors. Frequencies and percentages were calculated for summarizing categorical variables. Means and standard deviations were calculated for describing continuous variables. Chi-square test and *t*-test were employed to compare baseline information between prematurely terminated patients and methadone-maintained patients. Linear regression analysis was performed to examine influences of past retention on HRQoL, and significant independent variables are presented with confidence intervals. To control the confounding effects of baseline information, all variables of baseline information were included in the multivariate analysis. The general linear model was used to further identify the global effect of past treatment duration on HRQoL. Cox regression analysis was conducted to explore the influences of HRQoL on follow-up retention, and the values are presented with confidence intervals. In the Cox regression model, the event of interest was premature termination with time measured as days of MMT since April 1, 2012 until premature termination or the end of the study, whichever came first; individuals who remained on MMT through March 31, 2012, were considered censored. The baseline information and past retention were included as the controlling variables. All statistical analyses were performed using SPSS 22.0 (IBM Corp., Armonk, NY). A value of *P* < 0.05 (two-tailed) was considered statistically significant.

### Ethical statement

The study protocol was reviewed and approved by the Human Research Ethics Committee of Xi’an Jiaotong University. Written informed consent was obtained from each recruited patient before the questionnaire survey.

## Results

Of the 1,270 eligible patients, 58 (4.6%) patients (30 in the publicly funded clinic and 28 in the privately funded clinic) were excluded because they refused to provide written informed consent. Finally, a total of 1,212 patients completed the cross-sectional questionnaire survey at baseline, including 361 patients (29.8%) from the publicly funded clinic and 851 patients (70.2%) from the privately funded clinic. All patients were self-funded for methadone treatment, except for those with an HIV-positive serostatus. In the face-to-face interview, patients were informed about and understood the questions, and completed the questionnaire. Each interview lasted for approximately 20–25 min.

### Baseline information

The mean age at initial treatment was 39.24 ± 6.24 years (range: 19–69 years), and the total population included 934 (77.1%) men. The majority of the patients had available sociodemographic data for education level (secondary, n = 1076, 88.8%), marriage status (married, n = 694, 57.3%), and employment status (unemployed, n = 851, 70.2%). Age at initial drug use was 28.92 ± 7.70 (range: 12–66 years), 89.4% patients (n = 1083) underwent detoxification before initial MMT, and 64.9% patients (n = 786) used intravenous drugs. A small proportion of patients reported sharing syringe with peer drug users (n = 65, 5.4%) and a positive result for initial morphine urine test (n = 90, 7.4%) (Table 1).

**Table 1:** Description of sample at baseline and follow-up (*n*, %).

Baseline information	Baseline (N = 1212)	Two-year follow-up ^ƚ^		Statistics	*P*
		Premature termination (n = 527)	Maintain (n = 684)		
**Socio-demographics**					
Age at initial treatment (years) (mean ± SD) (range: 19–69)	39.24 ± 6.24	38.34 ± 6.41	39.93 ± 6.03	*t* = -4.44	<0.001
≤ 30	102 (8.4)	59 (11.2)	43 (6.3)	χ^2^ = 14.29	0.003
31–40	612 (50.5)	273 (51.8)	339 (49.6)		
41–50	460 (38.0)	184 (34.9)	275 (40.2)		
> 50	38 (3.1)	11 (2.1)	27 (3.9)		
Gender					
Male	934 (77.1)	404 (76.7)	529 (77.3)	χ^2^ = 0.13	0.72
Female	278 (22.9)	123 (23.3)	155 (22.7)		
Education level					
Primary and below	81 (6.7)	32 (6.1)	49 (7.2)	χ^2^ = 3.28	0.19
Secondary	1076 (88.8)	465 (88.2)	610 (89.2)		
Tertiary	55 (4.5)	30 (5.7)	25 (3.7)		
Marital status					
Married	694 (57.3)	292 (55.4)	401 (58.6)	χ^2^ = 1.26	0.26
Other marital status	518 (42.7)	235 (44.6)	283 (41.4)		
Employment status					
Unemployed	851 (70.2)	371 (70.4)	479 (70.0)	χ^2^ = 0.02	0.89
Employed	361 (29.8)	156 (29.6)	205 (30.0)		
**Drug use characteristics**					
Age at initial drug use (year) (mean ± SD) (range: 12–66)	28.92 ± 7.70	28.21 ± 7.50	29.46 ± 7.80	*t* = -2.80	0.005
≤ 20	189 (15.6)	102 (19.4)	87 (12.7)	χ^2^ = 11.17	0.011
21–30	532 (43.9)	220 (41.7)	312 (45.6)		
31–40	396 (32.7)	170 (32.3)	225 (32.9)		
> 40	95 (7.8)	35 (6.6)	60 (8.8)		
Detoxification before initial MMT					
Yes	1083 (89.4)	468 (88.8)	614 (89.8)	χ^2^ = 0.29	0.59
No	129 (10.6)	59 (11.2)	70 (10.2)		
Drug using methods over the past 6 months					
Oral	384 (31.7)	163 (30.9)	220 (32.2)	χ^2^ = 0.43	0.80
Injection	786 (64.9)	347 (65.8)	439 (64.2)		
Others	42 (3.5)	17 (3.2)	25 (3.7)		
Syringe Sharing experiences					
Yes	65 (5.4)	25 (4.7)	40 (5.8)	χ^2^ = 0.71	0.40
No	1147 (94.6)	502 (95.3)	644 (94.2)		
Initial morphine urine test					
Positive	90 (7.4)	48 (9.1)	41 (6.0)	χ^2^ = 4.24	0.04
Negative	1122 (92.6)	479 (90.9)	643 (94.0)		

MMT: methadone maintenance treatment.

^ƚ^ One patient lost to follow-up due to transferring to another clinic.

### Retention

Since the first admission, patients with repeat terminations accounted for 37.8% (n = 458) of the study population. Past treatment duration was 1057.37 ± 517.05 days (range: 1–1984 days) and categorized as follows: <1 year (n = 171, 14.1%), 1 year (n = 221, 18.2%), 2 years (n = 157, 13.0%), 3 years (n = 183, 15.1%), and ≥4 years (n = 480, 39.6%).

A total of 1,211 patients completed the 2-year follow-up, with 527 (43.5%) prematurely terminated patients and 684 (56.4%) methadone-maintained patients; one patient was lost to follow-up due to transfer to another MMT clinic. The follow-up treatment duration was 598.95 ± 207.12 days (range: 4–730 days): 25 patients maintained MMT for <1 month (2.1%), 34 patients maintained MMT for 1 month (2.8%), 33 patients maintained MMT for 3 months (2.7%), 125 patients maintained MMT for 6 months (10.3%), 111 patients maintained MMT for 12 months (9.2%), and 883 patients maintained MMT for ≥18 months (72.9%).

Significant differences in the baseline information between prematurely terminated patients and methadone-maintained patients were observed for age at initial treatment (*t* = -4.44, *P* < 0.001; χ^2^ = 14.29, *P* = 0.003), age at initial drug use (*t* = -2.80, *P* = 0.005; χ^2^ = 11.17, *P* = 0.011), and initial morphine urine test (χ^2^ = 4.24, *P* = 0.04); none of the other parameters differed significantly between the two groups (Table 1).

### HRQoL

In the SF-36v2, both the component summary and 8 scale mean scores were <50 points [24]. The two-component summary scores were 48.62 ± 7.94 for PCS and 41.02 ± 10.74 for MCS. The 8 scale scores were as follows: 49.83 ± 7.39 for physical functioning, 48.24 ± 10.86 for bodily pain, 46.93 ± 10.43 for vitality, 45.43 ± 10.48 for role physical, 44.52 ± 10.07 for social functioning, 41.66 ± 10.48 for mental health, 41.56 ± 12.01 for role emotional, and 39.74 ± 11.02 for general health. The QOL-DAv2.0 total score was 64.45 ± 17.48, with the 4 scale scores of 75.43 ± 20.61 for symptoms, 67.61 ± 24.01 for psychology, 56.99 ± 19.64 for physiology, and 56.97 ± 17.26 for society (data not tabulated).

### Relationships between past retention and HRQoL

Without considering the baseline information, patients with no terminations had higher SF-36v2PCS scores than those with repeat terminations (regression coefficient [B]: 1.33; 95% confidence interval [CI]: 0.41, 2.25; *P* = 0.005). Past treatment duration affected the SF-36v2PCS (*P* < 0.001) score and QOL-DAv2.0 (*P* = 0.032) score: The SF-36v2PCS (B: -3.15; CI: -4.53, -1.78; *P* < 0.001) and QOL-DAv2.0 (B: -3.87; CI: -6.91, -0.83; *P* = 0.013) scores for treatment for ≥4 years were lower than those for treatment for <1 year. After controlling the baseline information (the variables of baseline information included in the multivariate models were not strongly correlated), past treatment duration was found to influence the SF-36v2PCS (*P* = 0.004) scores: treatment for 3 years (B: -1.72; CI: -3.38, -0.05; *P* = 0.043) and ≥4 years (B: -2.39; CI: -3.80, -0.97; *P* = 0.001) showed lower SF-36v2PCS scores than treatment for <1 year (Table 2).

**Table 2:** Influences of repeated terminations and past treatment time on HRQoL: Linear regression analysis (N = 1212).

Independent variables	SF-36v2PCS		SF-36v2MCS		QOL-DAv2.0	
	B (95%CI)	*P*	B (95%CI)	*P*	B (95%CI)	*P*
**Univariate analysis**						
Repeated terminations						
Yes	0.00		0.00		0.00	
No	1.33 (0.41, 2.25)	0.005	0.35 (-0.90, 1.60)	0.58	1.86 (-0.17, 3.89)	0.07
Past treatment time (year)^a^		<0.001		0.31		0.032
< 1	0.00		0.00		0.00	
1~	-1.09 (-2.66, 0.48)	0.17	-1.23 (-3.37, 0.91)	0.26	-1.17 (-4.64, 2.31)	0.51
2~	-1.74 (-3.45, -0.04)	0.045	0.11 (-2.23, 2.43)	0.93	-0.16 (-3.95, 3.62)	0.93
3~	-2.26 (-3.90, -0.62)	0.007	-0.89 (-3.13, 1.35)	0.44	-3.15 (-6.78, 0.48)	0.09
≥ 4	-3.15 (-4.53, -1.78)	<0.001	-1.61 (-3.48, 0.27)	0.09	-3.87 (-6.91, -0.83)	0.013
**Multivariate analysis** ^**Ɨ**^						
Repeated terminations						
Yes	0.00		0.00		0.00	
No	0.76 (-0.18, 1.69)	0.11	0.09 (-1.19, 1.37)	0.89	1.17 (-0.89, 3.22)	0.27
Past treatment time (year)^b^		0.004		0.63		0.30
< 1	0.00		0.00		0.00	
1~	-0.76 (-2.33, 0.81)	0.34	-1.07 (-3.23, 1.09)	0.33	-0.76 (-4.22, 2.71)	0.67
2~	-1.64 (-3.36, 0.09)	0.06	0.20 (-2.17, 2.56)	0.87	-0.17 (-3.97, 3.64)	0.93
3~	-1.72 (-3.38, -0.05)	0.043	-0.52 (-2.81, 1.77)	0.65	-2.28 (-5.95, 1.40)	0.23
≥ 4	-2.39 (-3.80, -0.97)	0.001	-1.05 (-2.99, 0.90)	0.29	-2.46 (-5.59, 0.66)	0.12

^Ɨ^ Multivariate analysis was done after controlling following variables: age at initial treatment (year: ref. ≤ 30); gender (ref. male); education level (ref. primary and below); marital status (ref. married); employment status (ref. unemployed); age at initial drug use (year: ref. ≤ 20); detoxification before initial MMT (ref. yes); drug using methods over the past 6 months (ref. oral); syringe sharing experiences (ref. yes); and initial morphine urine test (ref. positive).

HRQoL: health-related quality of life. SF-36v2: short-form 36 health survey version 2. PCS: physical component summary. MCS: mental component summary. QOL-DAv2.0: quality of life scale for drug addicts. B: regression coefficient. 95%CI: 95% confidence interval.

^a^ *P*-value is for the test of effect of past treatment time on HRQoL in univariate analysis.

^b^ *P*-value is for the test of effect of past treatment time on HRQoL in multivariate analysis.

### Relationships between HRQoL and 2-year follow-up retention

To understand the HRQoL status, SF-36v2PCS, SF-36v2MCS, and QOL-DAv2.0 scores were categorized according to the corresponding median scores of 49, 42, and 66, respectively; patients with SF-36v2PCS scores > 49, SF-36v2MCS scores > 42, or QOL-DAv2.0 scores > 66 were considered to have better physical, psychological, or drug addiction-related HRQoL. Without considering the baseline information and past retention, SF-36v2PCS scores > 49 (hazard ratio: 0.83, 95% CI: 0.70, 0.99, *P* = 0.04) or SF-36v2MCS > 42 (hazard ratio: 0.84; 95% CI: 0.70, 0.99; *P* = 0.04) predicted a lower risk of premature terminations. On controlling for the baseline information and past retention (the variables of baseline information and past retention included in the multivariate models were not strongly correlated), we found that patients with an SF-36v2PCS score > 49 [hazard ratio (HR): 0.83, 95% CI: 0.69, 0.98] were 17% less likely to terminate MMT than those with an SF-36v2PCS score ≤ 49. However, the differences in premature termination were not significant between patients with SF-36v2MCS scores ≤ 42 and those with scores > 42, and between patients with QOL-DAv2.0 scores ≤ 66 and those with scores > 66 (Table 3).

**Table 3:** Effects of HRQoL on the risk of premature MMT termination: Cox regression analysis (N = 1211).

HRQoL	HR (95% CI)	*P*	HRQoL	HR (95% CI)	*P*
Univariate analysis			Multivariate analysis ^Ɨ^		
SF-36v2PCS^a^			SF-36v2PCS^a^		
≤ 49	1.00		≤ 49	1.00	
> 49	0.83 (0.70, 0.99)	0.04	> 49	0.83 (0.69, 0.98)	0.03
SF-36v2MCS^b^			SF-36v2MCS^b^		
≤ 42	1.00	≤ 42	1.00		
> 42	0.84 (0.70, 0.99)	0.04	> 42	0.84 (0.71, 1.00)	0.06
QOL-DAv2.0^c^			QOL-DAv2.0^c^		
≤ 66	1.00		≤ 66	1.00	
> 66	0.85 (0.72, 1.01)	0.06	> 66	0.86 (0.72, 1.03)	0.10

^a^ SF-36v2PCS was grouped by the median score 49.

^b^ SF-36v2MCS was grouped by the median score 42.

^c^ QOL-DAv2.0 was grouped by the median score 66.

Outcome (Y): premature termination = 1; MMT retention until the end of follow-up = 0.

Time (*t*): actual days of MMT from April 1, 2012 to March 31, 2014.

^Ɨ^ Multivariate analysis was performed after controlling variables assigned as follows: age at initial treatment (year: ref. ≤ 30); gender (ref. male); education level (ref. primary and below); marital status (ref. married); employment status (ref. unemployed); age at initial drug use (year: ref. ≤ 20); detoxification before initial MMT (ref. yes); drug using methods over the past 6 months (ref. oral); syringe sharing experiences (ref. yes); initial morphine urine test (ref. positive); repeated terminations (ref. yes); and past treatment time (year: ref. < 1).

HR: hazard ratio. 95%CI: 95% confidence interval. MMT: methadone maintenance treatment. HRQoL: health-related quality of life. SF-36v2: short-form 36 health survey version 2. PCS: physical component summary. MCS: mental component summary. QOL-DAv2.0: quality of life scale for drug addicts.

## Discussion

Although MMT has been considered an effective opiate-replacement therapy, retention in MMT is still suboptimal in most MMT programs [10,16,29]. Of the 1,212 patients, 37.8% had repeat terminations since initial treatment, indicating the high prevalence of repeated terminations in the MMT patient population; this is consistent with the findings of a related previous study [16]. After the 2-year follow-up, 43.5% patients terminated MMT: 27.1% patients quit MMT within the first 12 months, consistent with the findings of previous systematic reviews [11,14]. Thus, repeat terminations and early treatment discontinuations should be considered in MMT retention management and intervention programs.

This study showed that age at admission, age at first drug use, and initial urinalysis drug screen are predictors of premature discharge in MMT; consistent with this finding, previous studies have shown that young age at treatment, young age at initial drug use, and positive morphine urine test were related to high risks of early termination [30–33]. Therefore, age at admission, age at initial drug use, and pretreatment morphine urine test results should be considered when developing retention interventions, especially for an individualized management model for newly admitted MMT patients.

All the SF-36v2 mean scores were lower than the normal score of 50 points, demonstrating that MMT patients have a poorer health status than the general population [24]. The summary component scores and 8 scale scores demonstrated poorer overall mental health than physical health and specific impairment in general health, which is in line with findings of previous studies [13,34]. Relatively lower scores of QOL-DAv2.0 in the domains of society, physiology, and psychology indicate that damage in these health domains is common in MMT patients, which explains why MMT patients have poor HRQoL, especially impairment in physical and social function [27,35]. Thus, more efforts should be taken to improve the physical, psychological, and social health of MMT patients during treatment to achieve other objective therapeutic effects such as reduced rates of positive morphine urine tests and secret drug use while receiving MMT.

Univariate analysis showed that patients with no treatment terminations had higher SF-36v2PCS scores than those with repeat terminations, indicating that consecutive MMT improved physical HRQoL [15,19]. This finding supports our first hypothesis: Patients with repeat terminations had poorer HRQoL than those with no terminations. However, this is not the same as the findings of the multivariate analysis, i.e., repeated terminations had no significant impact on HRQoL scores in patients with MMT. This difference is probably related to the control of baseline information, especially the age at initial treatment (years), age at initial drug use (years), and initial morphine urine test, which further support the hypothesis that these variables had an influence on early discharge in MMT and should be carefully considered in MMT clinical practice.

In addition, patients with longer past treatment duration had lower SF-36v2PCS and QOL-DAv2.0 scores, especially those with past treatment duration for ≥4 years. However, past treatment time (year) had no significant influence on the QOL-DAv2.0 score in the multivariate analysis, indicating that the quality of life specific to drug addiction is not influenced by past treatment duration while controlling the baseline information. Nonetheless, our study showed that the HRQoL decreased with an increase in the treatment duration; this result is inconsistent with that of other reports [9,13,36] and contradicts our second hypothesis that longer past treatment time predicts better HRQoL. The observed decrease in the SF-36v2PCS and QOL-DAv2.0 scores is probably due to the following reasons: First, long-term methadone administration can lead to several potential side effects (e.g., weak or shallow breathing, severe constipation, dizziness, nausea, vomiting, increasing sweating, and sedation) [37], which might have a negative effect on the HRQoL. Second, MMT is effective in reducing heroin craving. However, some MMT patients are still at risk of craving heroin; these patients may relapse to heroin abuse, which may adversely affect their HRQoL [38,39]. Third, patients with syringe sharing are at high risk of HIV, HCV, or hepatitis B virus (HBV) infection that can lead to acquired immunodeficiency virus, chronic hepatitis C, or chronic hepatitis B, respectively, which would also deteriorate the HRQoL [34,40,41]. Therefore, the side effects of methadone should be carefully considered and addressed during long-term MMT. In addition, a morphine urine test should be performed regularly to supervise secret heroin use during MMT; other adjuvant medications for controlling or decreasing heroin craving are recommended on the basis of routine methadone prescription. Additionally, patients with positive HIV, HCV, or HBV serostatus should be treated accordingly, with the addition of methadone to their regimen, and syringe sharing should be strictly prohibited to avoid new HIV, HCV, or HBV infection.

Regarding the 2-year follow-up, patients with SF-36v2PCS scores > 49 (in both univariate and multivariate analyses) or SF-36v2MCS scores > 42 (in univariate analysis) had a lower risk of premature terminations than those with scores of ≤49 or ≤42, respectively, supporting our third hypothesis that better HRQoL predicts fewer treatment discontinuations; this result is consistent with the findings of a few previous studies [42,43], but contradicts the finding of another similar study that explored the HRQoL (measured by World Health Organization Quality of Life Assessment, Brief Version) on the basis of attendance rate among 105 self-funded heroin users and found that good social HRQoL was a predictor for poor methadone attendance [22]. This difference in our findings may result from the different characteristics of patients, study design, or analytical methods. Therefore, further study is required to predict the role of HRQoL in MMT retention.

Despite our important findings, this study had a few limitations. First, the baseline information did not include all variables; for example, subjective variables regarding psychological status were not included. Second, only a limited number of covariates were controlled for in the multivariate analysis; other unobserved factors such as religious beliefs and living conditions were not taken into account. Therefore, the findings may still be confounded by certain factors. Third, the results from this study reveal possible relationships between retention and HRQoL; however, they should not be interpreted as causal relationships, especially due to the relatively short follow-up period.

In conclusion, this study showed that patients with no terminations had better physical HRQoL than those with repeat terminations, and longer past treatment duration had a negative influence on physical and drug addiction-related HRQoL. Furthermore, better physical and psychological HRQoL was associated with a lower risk of premature terminations in MMT. This observed bidirectional relationship between retention and HRQoL should be considered when developing retention and health-management programs for MMT patients.
